# Sense of neighborhood belonging and health: geographic, racial, and socioeconomic variation in Wisconsin

**DOI:** 10.3389/fpubh.2024.1376672

**Published:** 2024-04-12

**Authors:** Joseph A. Clark, Michal Engelman, Amy A. Schultz, Andrew J. Bersch, Kristen Malecki

**Affiliations:** ^1^Center for Demography of Health and Aging, University of Wisconsin-Madison, Madison, WI, United States; ^2^School of Medicine and Public Health, University of Wisconsin-Madison, Madison, WI, United States; ^3^Division of Environmental and Occupational Health Sciences, School of Public Health, University of Illinois-Chicago, Chicago, IL, United States

**Keywords:** community, neighborhood, rurality, disparities, race

## Abstract

**Background:**

Individuals’ sense of belonging (SoB) to their neighborhood is an understudied psychosocial factor that may influence the association between neighborhood characteristics, health, and disparities across socio-demographic groups.

**Methods:**

Using 2014–2016 data from the Survey of the Health of Wisconsin (SHOW, *N* = 1,706), we conduct a detailed analysis of SoB and health in an American context. We construct OLS and logistic regressions estimating belonging’s association with general, physical, and mental health. We explore geographic, racial, and socioeconomic variation to understand both the differential distribution of SoB and its heterogeneous relationship with health.

**Results:**

A higher SoB is positively associated with better physical, mental, and general health. White participants report higher SoB than Black participants, yet the association between SoB and mental health is strongest among participants of color and urban residents.

**Conclusion:**

Sense of belonging to neighborhood significantly predicts many facets of health, with place and individual characteristics appearing to moderate this relationship. Racial, geographic, and socioeconomic disparities in belonging-health associations raise important questions about who benefits from the social, economic, and physical aspects of local communities.

## 1 Introduction

While a growing body of public health research focuses on the health-shaping effects of multiple physical and social contexts (1–6), less is known about the pathways through which macro-and meso-level factors influence individual health. Individuals’ experiences of social connectedness and integration are one important and policy-relevant pathway through which individuals come to embody contextual inequalities. Social belonging is a fundamental human need (7–9). At the individual level—social attachment and perceptions of local social cohesion have well-documented, positive associations with health (10, 11). Conversely, isolation, characterized by limited social support and fewer meaningful interactions, and feelings of loneliness have been consistently linked with higher mortality risk and a litany of morbidities (12). At the contextual level—such as the neighborhood, county, or state—social cohesion has been repeatedly linked with improved health and reduced mortality risk (13–17).

Sense of belonging (SoB) aims to characterize individuals’ assessment of how meaningfully they connect with others in a shared context (18). SoB is hypothesized to influence health through several psychosocial mechanisms. First, feelings of belonging have been argued to improve health by increasing individuals’ sense of control or agency (19, 20). Next, scholars consistently find negative associations between stress and SoB (21–26). Social belonging, as well as the material, informational, and emotional support of meaningful social connections reduce perceptions of stress and improve immune functioning (8, 27–31). Next, feelings of belonging and connectedness have been argued to improve self-esteem, which translates into better health behaviors and outcomes (1, 32, 33). Finally, belonging has been argued to buffer against social isolation and loneliness, which has been shown to be particularly deleterious for health (34). It is also worth noting that SoB may elicit changes in health-related behaviors and shape access to material and other forms of community resources (20).

Sense of belonging is a single-item, parsimonious measure of both individual and contextual characteristics (35). It is multi-dimensional in nature—capturing aspects of the micro-, meso-, and macro levels in which individuals are embedded. Belonging is part of the interstitial fabric of social life, and it sits along the pathway through which contexts become embodied in individuals (36). Both social relationships and community contexts influence individual health, and a sense of belonging to a community has likewise been linked with health status, life satisfaction, health behaviors, and other aspects of individual well-being (20, 37, 38). It is for perhaps this reason that experimental studies employing social-belonging interventions have pointed to SoB as a promising target for moderating the negative impacts of experiences of adversity (39) and increasing health equity (40–42). Still, key questions about potential heterogeneity in the relationship between SoB and health remain to be addressed if belonging is to play a role in understanding and improving public health in the United States: (1) What specific aspects of health are associated with SoB? (2) Do SoB-health associations hold in the United States? (3) Are these associations equally distributed across heterogeneous spaces and demographic groups?

The study of belonging and health is international. Building on concepts formalized by the field of community psychology, scholars from Canada, Australia, and Hong Kong have documented positive associations between SoB on self-rated health (21, 27, 32, 43). Despite this growing body of research, however, the study of belonging remains peripheral in United States public health research. This is conspicuous, given the non-trivial structural and social differences between the United States and other places where SoB research has been conducted. These include different migratory patterns, racial landscapes, governmental safety net and healthcare system structures, and built environments. All of these may affect individuals’ health, as well as their relationship with their communities. Although belonging is often alluded to in theorizing how social environments affect health in the United States (34, 44), it has not often been empirically engaged with and is accordingly understudied as a potential pathway to health, vulnerability, or resilience.

Because SoB is reflective of the relationship between individuals and their social contexts, it is sensitive to extraneous factors which shape the nature of those connections, such as racism and social heterogeneity, economic inequality, and spatial clustering. The meanings and impacts of belonging can be variable across groups and places. This perspective is supported by research on belonging interventions which demonstrated that individuals from groups which suffer from under-representation and stereotyping receive the greatest benefits (39–42). Similarly, researchers using data from the Canadian General Social Survey reported that associations between SoB and both self-rated and mental health were significant and positive among urban residents, but weaker or non-significant among rural residents (27). Given the translational concerns between the United States and international social landscapes outlined above, it is unclear for whom SoB matters the most for health.

Our study advances the literature using data collected by the Survey of the Health of Wisconsin (SHOW)—a unique, statewide representative sample of Wisconsin adults (45). SHOW’s comprehensive data and novel study design allows us to make four distinct contributions. First, we study the associations between belonging and health in greater detail than has been done before, incorporating numerous aspects of physical and mental health. Second, we examine these associations with a critical theoretical lens. Prior research has noted that associations between aspects of social capital and health outcomes may vary across subpopulations (46, 47). Therefore, we stratify our analyses by race/ethnicity, geographic, and socioeconomic factors to better understand variation in the pattern of SoB and its protective impact on health. Third, we add meaningfully to this area of research by studying participant-reported belonging to a geographical unit—one’s neighborhood. Finally, we conduct a detailed analysis of belonging and health in a United States context. Our findings underscore the importance to scholars and local stakeholders of understanding who benefits from the social, economic, and physical aspects of local communities and which populations are most impacted by shortcomings in these community characteristics.

## 2 Data and methods

### 2.1 Data

This study relies on a cross-sectional research design using 2014–2016 data from the Survey of the Health of Wisconsin (SHOW). SHOW is an annual, representative survey of civilian, non-institutionalized adults aged 21 and older in the State of Wisconsin (45). SHOW participants are recruited yearly from random households using a two-stage probability-based cluster sampling approach, stratified by region and poverty level. SHOW has response rates of 57.5, 63.5, and 85.6% at Waves I (2008–2013), II (2014–2016), and III (2017). Approximately 80% of participants who completed the household interview completed all survey components. By design, SHOW reflects a representative distribution across the entire spatial and sociodemographic range of the state population. While SHOW does contain follow-up surveys of some participants, SoB questions were only asked in follow-up survey participants’ most recent survey—at the same time that relevant health survey data was collected—restricting our ability to perform longitudinal analyses in this study.

Our analytic sample comprises 1,706 participants, including individuals from rural, suburban, and urban Wisconsin. Descriptive statistics for the study sample are provided in Table 1. A spatial distribution map of the SHOW sample is available in Appendix G.

**Table 1:** Descriptive statistics for the study sample.

Variable	Mean/Proportion	Std. Deviation
*Belonging*	-	-
Community belonging	3.69 (Range: 1–5)	0.82
*Health outcomes*	-	-
Mental health summary scores	51.07 (7.82–75.57)	10.11
Physical health summary scores	48.24 (9.99–69.24)	10.95
Stress scores	3.81 (0–19)	3.48
Fair/Poor self-rated health	0.13	0.33
Depression	0.24	0.43
Cancer	0.13	0.33
Diabetes	0.14	0.34
Hypertension	0.38	0.49
*Age*	-	-
Age at time of consent	51.22 (18–98)	18.05
Age 18–24	0.06	0.23
Age 25–34	0.16	0.36
Age 35–44	0.16	0.37
Age 45–54	0.15	0.35
Age 55–64	0.19	0.50
Age 65+	0.30	0.46
*Demographics*	-	-
White	0.83	0.38
Non-Hispanic Black	0.08	0.27
Other (Race)	0.05	0.22
Male	0.44	0.50
Married	0.59	0.49
*Geographic*	-	-
Rural	0.28	0.45
Urban	0.57	0.50
Suburban	0.16	0.36
*Socioeconomic status*	-	-
Income below poverty level	0.09	0.29
Income 100–200% poverty level	0.19	0.39
Income 200–400% poverty level	0.29	0.45
Income 400–600% poverty level	0.23	0.42
Income 600% + poverty level	0.20	0.40
Less than high school diploma	0.07	0.25
High school graduate (or GED)	0.23	0.42
Some college	0.36	0.48
Bachelor degree	0.23	0.42
Advanced degree	0.14	0.35

### 2.2 Outcome measures

To fully characterize the association between SoB and multiple dimensions of health, we consider a broad set of health measures including self-rated health, mental and physical health summary scores derived from the SF-12 Health Survey (48), and specific mental health and physical health indicators. Self-rated health is assessed in this study via a dichotomous measure of whether a participant categorizes their general health as “Good,” “Very Good,” or “Excellent.” Mental and physical health summary scores are constructed using the SF-12 Health Survey (48). Scores represent composite values which are converted to a 0–100 scale with a standard deviation of 10. Specific mental health domains are measured using self-reported symptoms of depression, anxiety, and stress scores constructed from participant responses to the Depression Anxiety Stress Scales 21-item questionnaire (DASS-21). States of depression, anxiety, and stress directly measure states of distress and are symptomatic of many of the most common mental health disorders (49, 50). Discrete physical health morbidities include measures of BMI, hypertension, diabetes, high cholesterol, and cancer also available in the SHOW.

### 2.3 Key predictors

Sense of belonging (SoB) in this study is measured using constructs of neighborhood belonging and is measured by participant responses to the prompt “I feel I belong in this neighborhood,” with possible responses ordered from “Strongly agree” to “Strongly disagree.” Recent work in Canada validated a single-item measure of belonging that was highly correlated with known neighborhood-level predictors of health, including: social capital, perceptions of crime and the built environment, and length of residence or “rootedness” (27, 35). Models are adjusted and subsequently stratified by control variables including age, race, sex, marital status, population density, income, and educational attainment. Population density is operationalized using Rural–Urban Commuting Area (RUCA) Codes 3 Category Classification, in which respondents are classified as living in a “urban,” “suburban,” or “rural” area (51). Race was measured using study participant self-classification, grouped for purposes of analysis into “Non-Hispanic White,” “Non-Hispanic Black,” or “Other.” The “Other” category concatenates the varied lived experiences of heterogeneous groups, but, issues of statistical power precluded more detailed comparisons. As such, within our analyses, “Other” is composed of Hispanic (any race), Asian, American Indian or Alaskan Native, Native Hawaiian or Pacific Islander, multiracial, and other (self-reported).

Prior studies have largely had to rely on survey questions asking participants about belonging to their community, broadly defined (21, 27, 43). Yet community may mean very different things to respondents of different backgrounds. It may be interpreted by respondents as one’s geographic neighborhood, an ethnic group, a religious tradition, or other relevant socially constructed categories. SHOW’s anchoring of SoB to one’s neighborhood allows us to gain more analytical clarity.

### 2.4 Analytic approach

**Figure 1:**
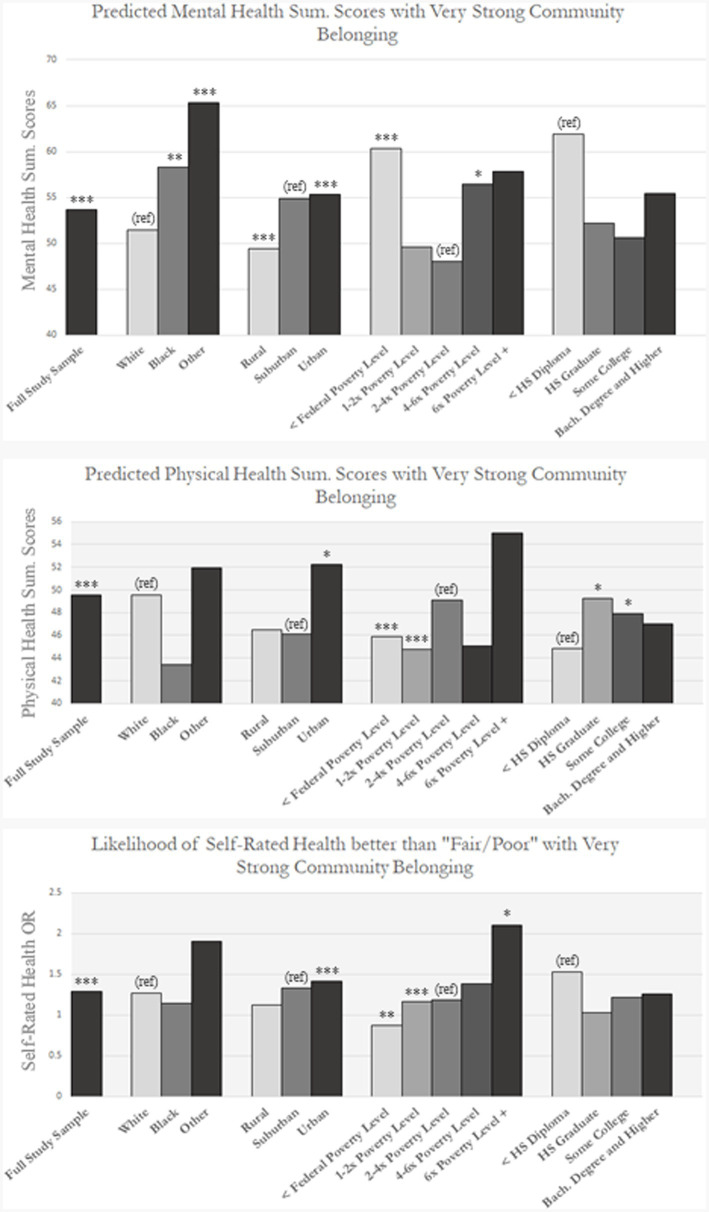
Predicted values within image are calculated using independent OLS and logistic regression analyses outlined previously in Tables 3, 4 and in Appendices B-D. Likelihood of self-rated health better than “Fair/Poor” is reported as an odds-ratio. Statistical significance indicators are based on models including interaction terms. ^*^p value < 0.1, ^**^p value < 0.05, and ^***^p value< 0.01.

All analyses include sampling weights to account for SHOW’s two-stage cluster study sampling design. Analyses were performed in STATA SE (16.1). To characterize the association between neighborhood SoB and a range of physical and mental health outcomes we first construct age, race, sex, and marital status adjusted OLS regression and logistic regression models (52, 53). Next, we stratify by age, race, population density, and socioeconomic status to generate predicted health scores and visualize variation in the SoB-health relationship. Finally, we construct interaction models to test the significance of these association across sociodemographic groups. Results of these analyses can be found in Figure 1, with predicted values from stratified models and significance indicators from the subsequent tests of interactions.

## 3 Results

Our SHOW sample consisted of 1,706 individuals, for which unweighted sample characteristics can be found in Table 1. Summaries of variation in sense of belonging (SoB) by race, education, income category, and geography can be found in Appendix A. Neighborhood SoB scores are consistent across urban, suburban, and rural areas, and show modest increases with education and income. There are moderate racial disparities in belonging, with White participants reporting somewhat higher average SoB (3.76) than Black participants (3.31).

Findings from regression analyses predicting health outcomes can be found in Table 2. We find that SoB is significantly protective of mental health summary scores (2.22; *p* < 0.001), physical health summary scores (0.89; *p* < 0.001) and self-rated general health (1.28 [Odds-ratio]; *p* < 0.01). We also examined subcomponents of mental health, including depression, anxiety, and stress scores from the DASS-21. Coefficients from these analyses can be found in Table 3. We find that greater sense of belonging is significantly associated with less depression (−0.96; *p* < 0.001), anxiety (−0.55; *p* < 0.001), and stress (−0.61; *p* < 0.001). These models reflect a consistency of association across mental health outcomes.

**Table 2:** Associations between sense of community belonging and mental health, physical health, and self-rated health.

Variables	Mental health sum scores		Physical health sum scores		Likelihood of good or better self-rated health	
	*b*	s.e.	*b*	s.e.	OR	s.e.
Sense of community belonging	2.22^***^	(0.35)	0.89^***^	(0.33)	1.28^**^	(0.14)
*Controls*	-	-	-	-	-	-
Age at consent	0.13^***^	(0.01)	−0.23^***^	(0.02)	0.99^**^	(0.00)
Black (Non-Hispanic)	−2.18	(1.37)	−4.03^***^	(1.54)	0.55^**^	(0.17)
Male	1.53^***^	(0.54)	0.97^*^	(0.55)	1.00	(0.17)
Married	1.71^***^	(0.57)	2.91^***^	(0.59)	1.95^***^	(0.34)
Intercept	34.23^***^	(1.52)	54.90^***^	(1.40)	3.53^***^	(1.41)
*R*-squared/Pseudo *R*-squared	0.13		0.14		0.03	

Adjusted odds-ratios are based on logistic regression models predicting the likelihood that show participants reported “Good,” “Very Good,” or “Excellent” general health. Beta coefficients are from OLS regression models predicting Mental Health and Physical Health Summary Scores from the SF-12.

^***^p < 0.01, ^**^p < 0.05, and ^*^p < 0.1.

**Table 3:** Associations between sense of community belonging and unstandardized mental health outcomes.

Variables	Mental health summary scores		Depression scores		Anxiety scores		Stress scores	
	(SF-12)		(DASS-21)		(DASS-21)		(DASS-21)	
	*b*	s.e.	*b*	s.e.	*b*	s.e.	*b*	s.e.
Sense of community belonging	2.22^***^	(0.35)	−0.96^***^	(0.15)	−0.55^***^	(0.10)	−0.61^***^	(0.14)
Intercept	34.23^***^	(1.52)	7.38^***^	(0.62)	4.74^***^	(0.45)	8.71^***^	(0.56)
R-squared	0.13		0.08		0.08		0.09	

Coefficients represent coefficients from regression analyses predicting aspects of mental health. Control variables included in models include age, sex, race, and marital status but are omitted from the table to preserve space.

^***^p < 0.01, ^**^p < 0.05, and ^*^p < 0.1.

In a similar manner, we examined the association of SoB with specific components of physical health (See Table 4). Here we found a less consistent picture. A higher neighborhood SoB was associated with better physical health summary scores (0.89; *p* < 0.001) and with 25% lower odds of diabetes. There were, however, no statistically significant associations with BMI, hypertension, blood cholesterol, or likelihood of cancer. These findings suggest a limited and conditional relationship between belonging and the physical health of Wisconsinites. Considered alongside the mental health results, they point toward a psychosocial mechanism linking belonging and health.

**Table 4:** Associations between sense of community belonging and unstandardized physical health outcomes.

Variables	Physical health summary scores		BMI		Hypertension		Diabetes		High cholesterol		Cancer	
	*b*	s.e.	*b*	s.e.	OR	s.e.	OR	s.e.	OR	s.e.	OR	s.e.
Sense of community belonging	0.89^***^	(0.33)	−0.42	(0.26)	1.05	(0.09)	0.75^***^	(0.07)	0.92	(0.08)	1.04	(0.12)
Intercept	54.90^***^	(1.40)	29.74^***^	(1.09)	0.01^***^	(0.00)	0.05^***^	(0.02)	0.02^***^	(0.01)	0.01^***^	(0.00)
*R*-squared/Pseudo *R*-squared	0.14		0.02		0.18		0.06		0.14		0.14	

Coefficients represent coefficients from regression analyses predicting aspects of mental health. Control variables included in models include age, sex, race, and marital status but are omitted from the table to preserve space.

^***^p < 0.01, ^**^p < 0.05, and ^*^p < 0.1.

To better understand potential disparities in these associations, we performed supplemental analyses for demographic, socioeconomic, and geographic subsamples of SHOW. Our findings are visualized in Figure 1, and specific coefficients can be found in Appendices B–D. In this figure, we also provide statistical significance indicators and reference categories for each association based on complimentary interaction term models. On the whole, the stratified analyses demonstrate that associations with belonging are not equally distributed racially, spatially, or socioeconomically. Specifically, we find that urbanites consistently have stronger associations between SoB and physical, mental, and self-rated health relative to their urban and suburban counterparts. While members of all racial groups have a significant and positive association between SoB and mental health, the associations are largest for members of self-identified Black and Other categories relative to their White counter parts. While White respondents also see significant positive associations between SoB and both physical and self-rated health (SRH), these relationships are not significant for respondents in the Black or Other categories. We note a nonlinear socioeconomic distribution of mental health associations with belonging, with a “U” shaped distribution in which those in the lowest and highest income categories had stronger associations than those in the middle. Levels of educational attainment associationally mirrored this pattern, though interaction terms were insignificant. By contrast, we find a somewhat linear and positive trend for income groups and SoB associations with physical health.

Interaction models confirm the significance of the subgroup differences described above. We report coefficients for these models in Appendix F. Statistically significant interaction terms were found between SoB and income across all three health outcomes, between SoB and race for mental health, and between SoB and urbanicity for mental and self-rated health. Our findings somewhat support the existence of a “U” pattern in mental health associations with SoB. By contrast, the trend for physical and self-rated general health appears linear, with the health-belonging association appearing to increase as income increases.

## 4 Discussion

The detailed health data in SHOW provide a richer picture of the SoB-health association than previous studies afforded. Using these data, we find significant and meaningful positive associations between neighborhood SoB and a broad span of health outcomes. The associations are stronger and more robust for mental health outcomes than physical health outcomes, and for urban residents relative to their rural and suburban counterparts. SoB is more consistently associated with health for White respondents, but there are particularly strong positive associations with mental health for respondents of color.

While we find significant associations between neighborhood SoB and physical health summary scores as defined by the SF-12 for some racial, geographic, and socioeconomic subgroups, we generally find the strongest and most consistent associations between SoB and mental health. This trend becomes even more stark when physical and mental health are disaggregated into specific morbidities—a unique insight available due to SHOW’s rich health measures. The strong associations with both overall and specific mental health measures mirror findings from Canadian scholars and provide support for theorized psychosocial mechanisms through which SoB influences health (27, 34). It is particularly suggestive of the buffering role that SoB may play in building up resilience. There is a notably strong association between sense of belonging and diabetes, though there is none for BMI. Future research should further explore potentially contributing mechanisms.

Importantly, we find that SoB-health associations are not evenly distributed across space, race, and socioeconomic status. Consistent with prior findings from Canada (27), we find that urban residents enjoy more positive associations for SoB and health than suburban or rural residents, across all measured health outcomes. Unlike previous research from Australia (32), however, we do not find a clear age trend or significant interactions between age and belonging for mental, physical, or self-rated health (see Appendix E).

Given the United States’ well-documented racial and socioeconomic disparities, our stratified analysis uncovers notable differences in the association between SoB and health. Self-identified Black participants have a stronger association between SoB and mental health relative to their White counterparts. This is reversed for physical health—with a stronger association with SoB for White participants. It is worth noting that Black Wisconsinites in SHOW report lower SoB on average than White Wisconsinites, suggesting that Black residents may feel less socially attached to their neighborhoods. Historical processes of segregation such as red-lining and economic disinvestment in communities of color may help explain the smaller association of physical health with belonging in these neighborhoods, as most of SHOW’s Black participants reside in urban neighborhoods. It is important to note that these findings are not the product of hypothesis testing, but are observational in nature. Models which do include interaction terms yield partial results, possibly due to limited statistical power, with significant interactions only within the mental health analyses.

The relationship between Socioeconomic disadvantage and SoB when predicting health is not always straight-forward. The positive association between SoB and Physical and self-rated general health seems to increase with income. This is complicated, however, by the non-linear relationship between SES and SoB in the case of mental health. Both the lower and upper tails of the income and educational distribution see the largest associations with mental health. One possible interpretation is that those with the lowest SES may find access to material and interpersonal resources and opportunities through local connections with others. Belonging may thus increase their resilience to material deprivation. Those whose low SES is accompanied by a low SoB are less likely to have these supportive networks and are unprotected against the well-documented weathering forces of inequality. At the same time, those with high SES who report a high SoB are more likely to reside in healthier built environments inhabited by others also of high SES. While their networks contain more people with greater resources, these respondents also live with resources and amenities that decrease their need to rely on these networks to the same extent. Our findings for high-SES participants may reflect this spatial clustering.

These findings raise the possibility that participants may interpret survey questions about neighborhood belonging differently depending on the intersection of personal characteristics, identity, and experiences and particular neighborhood contexts. There are well-documented material, cultural, and environmental differences in conditions between rural and urban areas in the United States (54, 55). Increased population density, improved walkability, clear spatial definitions of neighborhoods, and distinct patterns of segregation in United States cities produce very different lived experiences and expectations for urbanites. If neighborhood means something qualitatively different to urban and rural residents, perhaps this may explain some of why urban residents exhibit stronger relationships between belonging to neighborhood and health. Similarly, belonging may mean different things to rich and poor Americans. We found that the lowest and highest socioeconomic categories received the greatest premiums to belonging. It may be that lower income individuals in Wisconsin consider belonging from the perspective of local connections and support—vital to resiliency against acute stressors and material deprivation (56–58). Wealthier Wisconsin residents, on the other hand, may place greater stock in the structural and demographic components of the place, such as schools, resources, amenities, as well as racial and socioeconomic sameness. Understanding disparities in associations between belonging and health will require greater understanding of how individuals from different socioeconomic backgrounds assess and report belonging.

Much scholarship has examined the impact of social, economic, political, and built environments on individual and community health outcomes. Comparatively little research has concerned itself with whether and how individuals feel they belong and fit within these larger contexts and the emotional connection to place inherent in measures of neighborhood and community belonging. Public health researchers must more carefully consider fit between individuals and their environments when investigating social mechanisms that produce health disparities. We find that one aspect of fit—sense of belonging—is associated with better health, and that these associations are not equally shared. The findings in this study indicate that future research should consider belonging as an object in health research, as well as the relationships between belonging and individual and contextual characteristics. The high parsimony and portability of single-item belonging questions make them efficient and potentially high-return survey items (6). This makes SoB an excellent candidate for inclusion in existing, representative surveys—particularly those with large samples or longitudinal design. This research is a meaningful step toward better understanding SoB’s role in the health process. Ultimately, however, reproduction in different United States contexts is needed to more fully understand variation in SoB and its suitability for potential intervention.

Several limitations of our study are worth noting. The cross-sectional nature of this study limits our ability to infer causality, as our findings are subject to the possibility of confounding and reverse-causality. While this is not unique to our study, future research studying the relationship between SoB and health would benefit from longitudinal or experimental frameworks to address this issue. A recent intervention study demonstrated clear and compelling causal effects of belonging on college students’ mental health (39), providing some evidential support for the responsiveness of health to SoB. Still, broader validation is needed.

While SHOW data offers the opportunity to consider SoB in a racially diverse sample of participants across the rural/urban spectrum, future research on this topic would benefit from examining cohorts beyond Wisconsin. Our analyses additionally lack community-level variables such as those concerned with the natural or built environment, which may influence both health and belonging. This acts to limit the robustness of the study’s findings. Additionally, neighborhoods are clearly defined concepts in urban spaces and for urban residents, but are less accurate descriptions of the structure of rural environments. Thus, while previous research has consistently identified rural–urban differences in sense of belonging to “community” (21, 27) it is worth noting that our analyses may reflect contextual differences in how residents interpret the term “neighborhood.”

Finally, the positive correlations we find between belonging and health may be driven in part by worse health among the isolated or lonely. Investigating the full gradient between loneliness and belonging may be a useful and instructive topic for future research.

Despite these limitations, the study’s contributions are several. We produced a detailed study of neighborhood sense of belonging and health in a United States context. Our findings show that belonging matters for multiple health domains, and an analysis of the broadest range of health outcomes to date revealed particularly strong implications for numerous dimensions of mental health. We also uncovered both expected and unexpected geographic, demographic, and socioeconomic differences in these associations, suggesting that not everyone benefits equally from belonging, and that SoB may play different roles in different socioeconomic contexts. We argue that researchers should continue to investigate why and how belonging matters and for whom it matters the most.

## Data availability statement

The data analyzed in this study are subject to the following licenses/restrictions: data may be obtained from the Survey of the Health of Wisconsin and are not publicly available. Requests to access these datasets should be directed to Survey of the Health of Wisconsin, info@show.wisc.edu.

## Ethics statement

The studies involving humans were approved by University of Wisconsin Health Sciences Institutional Review Board (FWA00005399). The studies were conducted in accordance with the local legislation and institutional requirements. Written informed consent for participation was not required from the participants or the participants’ legal guardians/next of kin in accordance with the national legislation and institutional requirements.

## Author contributions

JC: Conceptualization, Formal analysis, Methodology, Writing – original draft, Writing – review & editing. ME: Conceptualization, Data curation, Funding acquisition, Methodology, Project administration, Writing – review & editing. AS: Data curation, Writing – review & editing. AB: Data curation, Resources, Writing – review & editing. KM: Data curation, Funding acquisition, Methodology, Project administration, Writing – review & editing.
